# The Cortical Motor System in the Domestic Pig: Origin and Termination of the Corticospinal Tract and Cortico-Brainstem Projections

**DOI:** 10.3389/fnana.2021.748050

**Published:** 2021-11-01

**Authors:** Patricia del Cerro, Ángel Rodríguez-De-Lope, Jorge E. Collazos-Castro

**Affiliations:** ^1^Neural Repair and Biomaterials Laboratory, Hospital Nacional de Parapléjicos, Toledo, Spain; ^2^Ph.D. Program in Neuroscience, Autonoma de Madrid University, Madrid, Spain; ^3^Department of Neurosurgery, Hospital Virgen de la Salud, Toledo, Spain

**Keywords:** pyramidal, corticospinal, porcine, pig, motor cortex, red nucleus, reticular formation, spinal cord

## Abstract

The anatomy of the cortical motor system and its relationship to motor repertoire in artiodactyls is for the most part unknown. We studied the origin and termination of the corticospinal tract (CST) and cortico-brainstem projections in domestic pigs. Pyramidal neurons were retrogradely labeled by injecting aminostilbamidine in the spinal segment C1. After identifying the dual origin of the porcine CST in the primary motor cortex (M1) and premotor cortex (PM), the axons descending from those regions to the spinal cord and brainstem were anterogradely labeled by unilateral injections of dextran alexa-594 in M1 and dextran alexa-488 in PM. Numerous corticospinal projections from M1 and PM were detected up to T6 spinal segment and showed a similar pattern of decussation and distribution in the white matter funiculi and the gray matter laminae. They terminated mostly on dendrites of the lateral intermediate laminae and the internal basilar nucleus, and some innervated the ventromedial laminae, but were essentially absent in lateral laminae IX. Corticofugal axons terminated predominantly ipsilaterally in the midbrain and bilaterally in the medulla oblongata. Most corticorubral projections arose from M1, whereas the mesencephalic reticular formation, superior colliculus, lateral reticular nucleus, gigantocellular reticular nucleus, and raphe received abundant axonal contacts from both M1 and PM. Our data suggest that the porcine cortical motor system has some common features with that of primates and humans and may control posture and movement through parallel motor descending pathways. However, less cortical regions project to the spinal cord in pigs, and the CST neither seems to reach the lumbar enlargement nor to have a significant direct innervation of cervical, foreleg motoneurons.

## Introduction

The artiodactyl forelimb is specialized for standing and locomotion. Among artiodactyls, pigs are even-toed ungulates with four digits in the forelimbs and hindlimbs. The first digit is absent in adult pigs, the second and fifth are vestigial and directed backward, whereas the third and fourth digits bear the body weight (Sears et al., 2011). The foreleg has no clavicle and has limited rotation, which allows for better walking stability but constrains other type of movements. Pigs usually perform skilled motor acts (for instance searching food) with the snout instead than with the forelimb (Goursot et al., 2018). However, pigs can still execute target-directed movements such as lever press maintenance with the forehoof and using the forelimb can be preferred over the snout in a context-dependent manner (Ferguson et al., 2009). In other mammals, the cerebral cortex is involved in the initiation of volitional movements and the selection of motor commands by selectively filtering sensory information and by coordinating posture with the activation of proximal and distal limb muscles (Canedo, 1997). Therefore, it is expected that the porcine cortical motor system has a similar role regarding purposeful motor behavior. However, there is a gap of knowledge regarding the forelimb motor repertoire in swine and the precise neural basis for its volitional control.

In primates, cats, and rodents, cerebral cortex commands reach spinal neurons directly through the corticospinal tract (CST) and indirectly via cortical projections to several brainstem nuclei, particularly to the magnocellular red nucleus (mRN), the superior colliculus (SC), and the ponto-medullary reticular formation (Kuypers, 1962; Armand, 1982; Canedo, 1997), which give rise to the rubrospinal (RuST), tectospinal (TST), and reticulospinal (RST) tracts, respectively. Besides, corticospinal axons may drive the spinal motor output through disynaptic pathways involving propriospinal neurons and segmental interneurons (Dum and Strick, 1996; Alstermark et al., 1999; Yoshino-Saito et al., 2010; Isa et al., 2013), or through monosynaptic cortico-motoneuronal connections, which are prominent in humans and some non-human primates (Bernhard and Bohm, 1954; Phillips and Porter, 1964; Heffner and Masterton, 1975; Porter, 1985; Yoshino-Saito et al., 2010; Lemon, 2019) and allow executing volitional movements that demand fractionated limb coordination and high manual dexterity (Lawrence and Kuypers, 1968; Heffner and Masterton, 1983; Porter, 1985; Duque et al., 2003; Gu et al., 2017).

Anatomical and physiological data for the cortical motor system have been extensively reported for rodents (Terashima, 1995; Brösamle and Schwab, 2000; Collazos-Castro et al., 2006; López-Dolado et al., 2013; Moreno-López et al., 2016; Ueno et al., 2018; Steward et al., 2021), cats (Hern et al., 1962; Illert et al., 1977; Groos et al., 1978; Alstermark et al., 1991; Canedo and Lamas, 1993; Ghosh, 1997; Salimi and Martin, 2004; Jankowska et al., 2006), and primates (Kuypers, 1962; Phillips and Porter, 1964; Dum and Strick, 1991, 1996, 2002; Galea and Darian-Smith, 1994; Rosenzweig et al., 2009; Alstermark et al., 2011; Witham et al., 2016), whereas little information is available for pigs despite their increasing use in neurological research. Earliest retrograde neural tracing studies injected horseradish peroxidase (HRP) into the cervical and lumbar spinal cord, finding several cortical regions with spinal projections in cats and monkeys (Coulter et al., 1976; Groos et al., 1978). After, a more complete identification of the cortical origin of corticospinal axons was accomplished in 22 species of mammals, including primates and rodents but not swine, by applying HRP after a C1-C2 spinal cord hemisection (Nudo and Masterton, 1990). Corticospinal neurons were found only in cortical layer V intermingled with pyramidal neurons not projecting to the spinal cord. At least two regions of neocortex contributed corticospinal fibers in those species, and a third segregated region was present in primates, some rodents, and lagomorphs. Subsequent studies confirmed that several cortical regions send CST axons in primates (Dum and Strick, 1991; Galea and Darian-Smith, 1994; Luppino et al., 1994; Strick et al., 2021). In fact, neurons from at least six areas of the primate frontal cerebral cortex project to both the primary motor cortex and the spinal cord. Each corticospinal neuron population participates in distinct neural circuits with the basal ganglia and cerebellum and is differentially connected at the spinal level, thus subserving specific aspects of motor behavior (Galea and Darian-Smith, 1994; Dum and Strick, 2002; Strick et al., 2021).

The localization of the porcine motor cortex was initially mapped by electrical stimulation through epicortical electrodes in anesthetized pigs, identifying a well-defined motor area in the cruciate gyrus (Breazile et al., 1966; Palmieri et al., 1987). This cortical region is homologous to the precentral gyrus of primates and will be hereafter referred to as M1. Studies on axonal Wallerian degeneration ensuing M1 lesion in domestic pigs led to the conclusion that the porcine CST was essentially inexistent, the pyramidal tract consisting only of cortico-bulbar axons that innervated cranial motoneurons and ended at the pyramidal decussation (Palmieri et al., 1987). This notion prevailed for two decades until it was re-addressed by injecting anterograde neural tracers in M1 (Leonard et al., 2017), showing that porcine CST axons travel along the complete cervical spinal cord. Also using anterograde tracers, Bech et al. (2018) found that about 86% of the porcine CST fibers crossed in the pyramidal decussation and descended by the contralateral dorsolateral fasciculus of the upper cervical spinal cord, and suggested that the premotor cortex may contribute CST axons.

In the present study, we used retrograde and anterograde fluorescent neural tracers to investigate the cerebral cortex regions that project corticospinal axons in domestic pigs, and the termination of those axons within the spinal gray matter. We also investigated the innervation of brainstem nuclei by the identified porcine cortical motor areas.

## Materials and Methods

### Animals

Five Large White male pigs (*Sus scrofa domesticus*), purchased from a commercial supplier (Granja Agropardal, Toledo, Spain), were used in this study. The experimental protocols adhered to the recommendations of the European Commission and Spanish regulations for the protection of experimental animals (86/609/CEE, 32/2007 and 223/1988) and were approved by the Ethical Committee for Animal Research of the Hospital Nacional de Parapléjicos. Pigs 1 and 2 (22-weeks-old, 35 kg and 38 kg, respectively) received injections of retrograde neural tracer in the right side of the C1 spinal segment, whereas pigs 3 (7-weeks old, 16 kg), 4 (11-weeks old, 17 kg), and 5 (22-weeks old, 30 kg) received injections of anterograde neural tracers in the left cerebral cortex. Pigs 1 and 2 were killed fifteen days after tracer injection, i.e., when the animals were about 6 months old. Pigs 3, 4, and 5 were killed 2 months after tracer injection, i.e., when the animals were approximately 4, 5, and 7 months old, respectively. The 2-month survival period was aimed at allowing both anterograde tracer transport and further maturation of neural connectivity (see “Discussion” section). Moreover, for anterograde CST tracing, using animals of different size allowed us to rule out that insufficient tracer transport prevented labeling of corticospinal axons in the caudal-thoracic and lumbar spinal cord (see “Results” section).

### Surgical Procedures

All surgical procedures were performed under aseptic conditions and inhalational anesthesia. Anesthesia was induced by intramuscular (IM) injection of ketamine (10 mg/kg), midazolam (0.1 mg/kg), and medetomidine (0.02 mg/kg); followed by intravenous (IV) administration of propofol (3 mg/kg). Then, a tracheal tube was placed, and the anesthesia was maintained with sevoflurane (1.7–2%) together with remifentanil (26 mg/kg/h IV) and rocuronium (1.2 mg/kg/h IV). Mechanical ventilation (Fabius Tiro, Dräger) was set at 12–14 breaths/min with a tidal volume of 10–15 ml/kg. Heart rate, blood pressure, exhaled carbon dioxide, blood oxygen saturation, and inspired and expired sevoflurane levels were monitored during the procedure (Dräger Infinity Delta). Postoperatively, the animals received meperidine (4 mg/kg) subcutaneously (SC) each 12 h for 2 days for pain, marbofloxacin (2 mg/kg IM) as antibiotic for 7 days, and meloxicam (0.2 mg/kg SC) as anti-inflammatory agent.

#### Retrograde Tracing of Corticospinal Neurons

**FIGURE 1:**
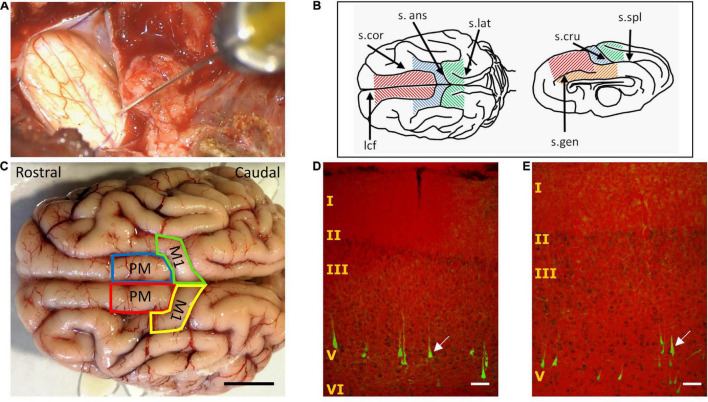
Retrograde tracing of corticospinal neurons (CSNs). **(A)** Intraoperative photograph of retrograde tracer microinjection in the caudal, mid-lateral region of the C1 spinal cord segment. **(B)** Drawing of Sus Scrofa Domestica telencephalon showing the regions analyzed in search of retrogradely-labeled CSNs. Frontal superior gyrus, red; cruciate gyrus, blue; rostral striate cortex, green; cingulate cortex, orange. *s.cor*, coronal sulcus; *s.ans*, ansate sulcus; *s.lat*, lateral sulcus; *lcf*, longitudinal cerebral fissure; *s.cru*, cruciate sulcus; *s.spl*, splenial sulcus; *s.gen*, genual sulcus. **(C)** Dorsal aspect of the pig cerebral cortex, delineating the areas in which retrogradely-labeled CSNs were found, namely the putative premotor cortex (PM) and primary motor cortex (M1). Scale bar, 1 cm. **(D,E)** Illustrations of CSNs labeled in layer V of M1 **(D)** and PM **(E)**, generated by merging transmitted light (for visualizing Cresyl violet) and fluorescence images (for aminostilbamidine), captured using a 10X objective for including most of the cerebral cortex in the field view. The arrows signal typical traced neurons with pyramidal shape and apical dendrites. Scale bar, 100 μm.

Maximal retrograde labeling of corticospinal neurons in primates and rodents was obtained by applying neural tracers to the cut axonal fibers after a high cervical spinal cord hemisection (Nudo and Masterton, 1990). However, we have shown that pigs with cervical C5-C6 spinal cord hemisection develop severe postural and motor deficits and become demanding for health care and basic behaviors (Cerro et al., 2021). Because those impairments are expected to be even greater after C1 hemisection, in the present work we attempted a less traumatic while still efficient method for retrograde neuronal tracing, namely injecting a relatively large total volume (48 μl) of the fluorescent tracer aminostilbamidine into the lateral funiculus of the right, caudal part of C1, at the approximate location of the dorsolateral component of the CST. In brief, the neck of the animals was disinfected with povidone-iodine and a 10-cm dorsal midline incision was made between the occipital bone and the C4 vertebra. Subsequently, the C1-C4 spinous processes were exposed and dorsal laminectomy of C1, followed by midline durotomy, were performed to access the C1 spinal segment. Afterward, a total of 48 μL of aminostilbamidine (40 mg/ml, Sigma-Aldrich) was injected in 12 points (4-μL each, at 2 mm lateral from the midline, at 2 mm and 4 mm of depth in the dorsoventral plane, spaced 5-mm longitudinally) using a 50 μL Hamilton syringe mounted in a micromanipulator (Figure 1A). The needle was set in place for 10 min to prevent reflux of the injected solution. The dura mater and the muscle planes and skin were separately sutured.

Aminostilbamidine differs from hydroxystilbamidine (Fluoro-Gold) only in an amino group substituting for a hydroxyl group, but otherwise these molecules are identical. The use of aminostilbamidine in the present work was based on our previous studies with this neural tracer in rodents (Collazos-Castro et al., 2005; Lucas-Osma and Collazos-Castro, 2009; López-Dolado et al., 2013) and pigs (Cerro et al., 2021) showing that: (1) it produces an intense, durable, and bleach-resistant fluorescent neuronal labeling in paraformaldehyde-fixed tissue; (2) it is suitable for tracing neurons with long axons and can be easily combined with Alexa-conjugated dextran and other tracers for multiple cell labeling; (3) neuronal uptake of aminostilbamidine is very efficient and occurs by multiple ways including healthy axonal terminals and apparently fibers of passage, as well as by axons damaged by the needle or tissue necrosis at the injection site, similarly to Fluoro-Gold (Schmued and Fallon, 1986; Dado et al., 1990).

#### Anterograde Tracing of Axonal Projections From the Motor Cortex to the Brainstem and Spinal Cord

After retrograde identification of M1 and the frontal premotor cortex (PM) as the areas that originate porcine corticospinal axons, anterograde neural tracers were injected in those areas in three additional animals. For this, a craniotomy was performed on the left side of the skull over the cruciate and the superior frontal gyri, removing the bone from the midline to about 3 cm lateral, and from 3.5 cm rostral to 0.5 cm caudal to the frontal-parietal suture. Two different tracers were injected to study the terminations of axons arising from each cortical region. Alexa 594-conjugated dextran (10,000 MW, Thermo Fisher Scientific Inc., D22913) was injected in M1, and Alexa 488-conjugated dextran (10,000 MW, Thermo Fisher Scientific Inc., D22910) was injected in PM. Both tracers were dissolved at 10% in saline solution. Twelve injections (4-μL each, at 2 mm and 4 mm of depth in the dorsoventral plane, separated 0.8-mm along the longitudinal axis of the respective cortical gyrus) of each tracer were made using a 50-μL Hamilton syringe mounted in a micromanipulator. The needle was set in place for 4 min to prevent reflux of the injected solution. The same total dose of 48 μL of each tracer was administered in all animals. The dura matter and scalp were closed separately.

#### Brain and Spinal Cord Extraction and Processing

The animals were deeply anesthetized as described above and the complete spinal cord was exposed by a dorsal surgical approach. Then, the pigs were euthanatized with pentobarbital (120 mg/Kg IV) and the complete spinal cord was quickly removed and immersed in 4% paraformaldehyde (PFA) dissolved in 0.1 M, pH 7.35 phosphate buffer. Subsequently, the skull was opened using an oscillating saw and the brain was also removed and immersed in PFA. After 3 days in the fixative, the brain and the spinal cord were dissected for analysis. Each spinal cord segment was identified by its dorsal and ventral roots and transversely cut into two portions (rostral and caudal). In pigs 1 and 2, the frontal superior gyrus, cruciate gyrus, rostral striate cortex, and cingulate cortex (Figure 1B) were processed separately for a precise mapping of the fluorescently labeled cells. In pigs 3, 4, and 5, the whole motor cortex was processed as a single block to verify the tracer injection sites. For all pigs, the brainstem was separated from the brain and cerebellum, placed in horizontal position, and divided longitudinally into portions of 5-mm in length. Each tissue block was cryoprotected by immersion in 30% sucrose in 0.1 M, pH 7.35 phosphate buffer for 4 days, and after was embedded in Optimal Cutting Temperature (OCT) and frozen at −20°C until cut in a cryostat.

### Tissue Sampling, Histological Procedures, and Cell Quantifications

#### Retrograde Neural Tracing With Aminostilbamidine

C1 spinal segment from pigs 1 and 2 was transversally cut at 50-μm thickness to investigate the appearance of the injection sites at fifteen days after tracer injection. Each fourth section was stained with Eriochrome cyanine for assessment of the spinal cord damage, and adjacent non-stained sections were employed to visualize aminostilbamidine with a fluorescence microscope.

For assessment of retrograde labeling of corticospinal neurons (CSNs), the entire cruciate gyrus, frontal superior gyrus, rostral striate cortex, and cingulate cortex from both brain hemispheres were cryosectioned at 50-μm thickness in the coronal plane. Cresyl violet staining was employed as a counterstain for the neuronal somas labeled with aminostilbamidine, thus allowing a reliable identification of their position in the cerebral cortex when simultaneously visualized with transmitted light and fluorescence microscopy (Figures 1D,E). For a qualitative assessment of the distribution of CSNs in each cortex, the fluorescent neuronal profiles were counted at intervals of 200 μm with an Olympus BX51 microscope and a 20X objective.

For assessment of retrogradely labeled brainstem neurons, the whole red nucleus (RN, ∼3 mm in rostrocaudal length) and the medulla oblongata, where the gigantocellular reticular nucleus (Gi) is located, were cryosectioned transversally at 50-μm thickness. Each fourth section was stained with Toluidine blue and the neural structures were identified with help of a porcine CNS atlas (Félix et al., 1999) and a human CNS atlas (Mai and Paxinos, 2012). Adjacent sections were used without further staining to visualize the aminostilbamidine fluorescence with an Olympus IX83 microscope equipped with a digital camera (Orca-Flash 4.0) and controlled by the CellSens Dimension software. Mosaic images of the Gi and the magnocellular RN were acquired with a Leica confocal microscope (TCS SP5) using a 20X objective.

#### Anterogradely-Labeled Corticospinal Axons

Corticospinal projections were labeled by injecting anterograde tracers in M1 and PM of pigs 3, 4, and 5. The motor cortices from the three animals were cryosectioned at 50-μm thickness in the coronal plane and subsequently stained with Toluidine blue to verify the placement of the injections.

Initially, we quantified corticospinal axons fluorescently labeled in the white matter (WM) of C2, C5, C8, T3, T6 and T7 spinal segments of the three pigs. For this, the rostral portion of the segments was horizontally cut at 50-μm thickness in a cryostat and mounted with Inmu-mount^TM^. Cutting the spinal segments at 50-μm thickness in the dorsoventral plane produced between 90 and 140 tissue sections depending on the segment. All sections were first inspected in a fluorescence microscope with a 20X objective to find the first and the last one of the series containing fluorescently labeled axons. Then, mosaic images of every other tissue section (i.e., at 100-μm intervals, ∼60 tissue sections imaged per segment and ∼360 sections imaged per spinal cord) were captured using a Leica DMI6000B microscope equipped with a digital camera (Leica DFC 350 FX) and a 20X objective (0.4 numeric aperture, 3.8-μm depth of field). A mosaic was composed of 6–10 images spanning the entire left-to-right aspect of the spinal cord at different dorsoventral depths. A shrinkage between 20 and 33% was measured in the thickness of the sections; therefore, each image comprised 11–14 serial optical sections (z-stack) spaced 3-μm in depth, thus assuring no loss of information because all fluorescent axons appeared in focus at least once in the z-stack. The axons intersecting a line perpendicular to the longitudinal axis of the spinal cord were counted in all optical sections of the z-stack using the Image J cell counter plugging for axon enumeration and prevention of double counting. All images in the mosaic were quantified in all the imaged tissue sections (i.e., half of those containing labeled axons, or ∼60 sections per segment), meaning that the reported values represent approximately half the real number of CST axons labeled at the respective segment in each animal. Axonal numbers are presented separately for three regions in each side, namely the dorsolateral (DLF), dorsomedial (DMF), and ventromedial (VMF) funiculi.

We performed a gross qualitative estimation of the innervation of the gray matter (GM) by corticospinal axons in the spinal segments C2, C5, and C8. For this, the caudal regions of those segments were transversally cut at 20-μm thickness, and three sections per segment were processed for MAP2 immunohistochemistry to label the spinal neuronal somas and dendrites. The tissue sections were unmasked for antigen retrieval by heating at 90°C for 25 min with 0.01 M sodium citrate; after, they were blocked for 1 h in PBS containing 1% triton and 2% normal donkey serum, rinsed three times with PBS, and incubated overnight at 4°C with mouse anti- Microtubule-associated protein 2 (MAP2; Sigma M1406; 1:500), diluted in phosphate buffer with 1% triton and 1% normal donkey serum. The sections were then washed with phosphate buffer and incubated for 2 h with donkey secondary fluorescent antibody (Cyanine Cy5 anti-mouse; Jackson Immuno Research 715-175-150; 1:100) at room temperature. MAP2 labeling allowed us to delineate the regions of interest within the GM, namely the internal basilar nucleus (IB), and the intermediolateral (IL), ventromedial (VM), and ventrolateral (VL) areas. Because the IB is in the ventromedial dorsal horn, within the limits of lamina IV of C1-C6 segments, the innervation in that nucleus was measured only in C2 and C5 segments. A mosaic of ∼0.216 mm^2^ was acquired for each region in a given section using a confocal microscope (Leica TCS SP5) with a 63X objective. The images were processed with the Image J software. A size threshold was applied to exclude fluorescent particles bigger than 40 μm^2^, which corresponded to perivascular cells and dust, thus obtaining a clean signal from axonal branches and varicosities. The area occupied by this signal was measured and expressed as percentage of the total analyzed area. The resulting value was further divided for the number of corticospinal axons counted at the WM of C2, to avoid bias arising from a different neuronal uptake of the tracer at the cerebral cortex in each animal. The number of axons labeled at upper CNS regions is widely used for normalizing neuroanatomical data resulting from anterograde tracing of motor cortex projections to the brainstem and spinal cord (Starkey et al., 2012; Weishaupt et al., 2014; Jin et al., 2015; Fregosi and Rouiller, 2017; Fregosi et al., 2019). That normalizer was also useful for our objective of estimating the relative innervation of brainstem nuclei and spinal cord segments by corticofugal axons from M1 and PM. In our study, besides some inevitably variation of size and precise location of tracer injections in the cerebral cortex, the specific anatomy of M1 and PM, the physicochemical properties of the used dextran conjugates, and possible differences in brain size between animals led to variable tracer transport by cortical projection neurons. Nevertheless, normalizing to axonal counts in C2 allowed us to correct most of these biases, thus producing comparable data sets (see “Results” section).

The specificity of the MAP2 antibody for staining dendrites and not axons was tested by performing a double immunohistochemistry for MAP2 (Sigma M1406; 1:500) and neurofilament (NF, Biomol NA1297; 1:400). Spinal cord sections from a healthy animal without any tracer applied were used to avoid confounding results in the observed fluorescence. Alexa Fluor anti-mouse 594 (Thermo Fisher Scientific A-11005, 1:1,000) and Alexa Fluor anti-rabbit 488 (ThermoFisher Scientific A-11008, 1:1,000) were used as secondary antibodies for MAP2 and NF, respectively.

As a first step in identifying spinal synaptic contacts by CST axons, immunohistochemistry for the postsynaptic density protein (PSD-95) was additionally performed in transverse sections of the C3 segment of pig 3. 16-μm-thick sections were unmasked for antigen retrieval by heating at 90°C for 20 min with 0.01 M sodium citrate; after, they were blocked for 1 h in PBS containing 0.2% triton and 2% normal donkey serum, rinsed three times with PBS, and incubated overnight at 4°C with rabbit anti-PSD-95 (Invitrogen 51-6900; 1:100), diluted in phosphate buffer with 0.2% triton and 1% normal donkey serum. The sections were then washed with phosphate buffer and incubated for 2 h at room temperature with donkey anti-rabbit secondary fluorescent antibody (DyLight 405; Jackson ImmunoResearch 711-475-152; 1:100).

#### Brainstem Innervation by Motor Cortex Axons

To assess the innervation of the midbrain and medulla oblongata by axons from M1 and PM, large mosaic images (∼3 cm^2^ and ∼1.5 cm^2^ for the midbrain and medulla oblongata, respectively) of 50-μm transverse tissue sections, at 600-μm intervals, were captured with the Olympus IX83 microscope and Orca-Flash 4.0 digital camera controlled by CellSens Dimension software. Adjacent tissue sections were processed for Toluidine blue staining to identify the regions of interest (ROIs). The red nucleus (RN), mesencephalic reticular formation (mRt) and superior colliculus (SC) were analyzed in the midbrain, whereas the raphe nuclei (R), gigantocellular reticular nucleus (Gi), and lateral reticular nucleus (LRt) were studied in the medulla oblongata. After drawing the ROIs in the mosaic images, brainstem innervation by corticofugal axons was estimated using the same protocol described for cortical innervation of the spinal cord.

### Statistics

All values reported, unless otherwise stated, are individual data for each pig or the mean ± standard error of the mean for the three pigs with anterograde tracers. As indicated in the text, some values were divided by the number of CST axons found in the WM at C2 of each animal, with the aim of reducing the possible bias caused by variability in the uptake of neural tracers. Student’s *t-*test was used for comparisons of axonal counts from M1 and PM.

## Results

### Retrograde Tracing of Encephalic Neurons Projecting to the Spinal Cord

The regions showing aminostilbamidine-labeled neurons were similar in pigs 1 and 2. Fifteen days after injecting the tracer in C1, CSNs were found in the cruciate gyrus (putative M1) and the frontal superior gyrus (putative PM) of both brain hemispheres (Figures 1B,C). No fluorescent cells were detected in the cingulate and the rostral part of the striate cortex. In both M1 and PM, more than 90% of CSNs was concentrated in a length of 10–15 mm in the most caudal part of the gyri (Figure 1C), while the remaining 10% was scattered in a few millimeters rostral to this region. Fluorescent CSNs were detected exclusively in layer V and had a pyramidal shape with apical dendrites (Figures 1D,E). Analyzes of the injection sites at C1 of pig 1 showed that the tracer was mostly confined to the targeted (right) side of the spinal cord, spreading in both the GM and WM (Supplementary Figure 1A). Neural damage ensuing tracer injections in this animal involved the right GM and WM regions containing CST axons, namely the DLF, DMF, and VMF (section “Distribution of Corticospinal Axons in the Spinal White Matter”), and extended to the left VMF and medial GM, as visualized in Eriochrome cyanine-stained sections. Intense aminostilbamidine fluorescence was detected in the right lateral and ventral WM, while the left lateral WM had healthy appearance and no fluorescent staining (Supplementary Figure 1A). Likely not all cortical neurons sending axons through the right side of the spinal cord were retrogradely labeled in pig 1 (see “Discussion” section); however, the neural tracer was certainly available to most of them in the right GM and the WM. On the other hand, the injections were more centrally placed in pig 2, causing substantial pathology in the GM and VMF bilaterally (Supplementary Figure 1B), with less tissue damage and tracer signal in the right lateral WM compared to pig 1. Because of the bilateral involvement of the GM, more labeled cells were evident in both brain hemispheres in pig 2. Therefore, we quantified the CSNs only in pig 1 to know the approximate contribution of each cortex and hemisphere to the CST. In this animal, a total of 5,440 CSNs were counted when analyzing 1 out of 4 tissue sections. 81.82% of CSNs was found in the contralateral (left) hemisphere (38.18% in M1 and 43.66% in PM), and 18.18% was in the ipsilateral (right) hemisphere (11.18% in M1 and 6.98% in PM).

**FIGURE 2:**
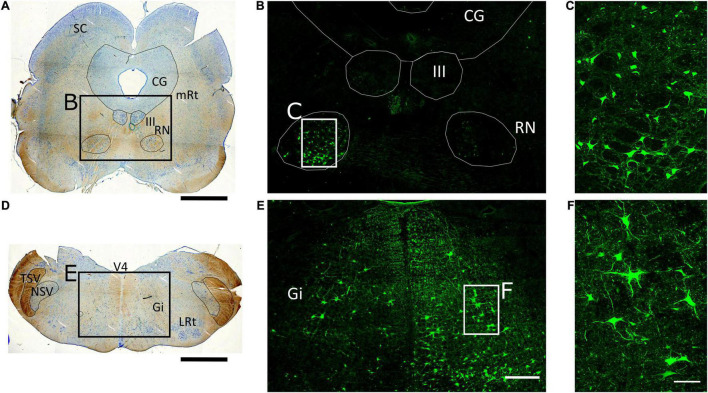
Retrograde tracing of brainstem nuclei projecting to the spinal cord. **(A,D)** 50-μm thick tissue sections of the porcine midbrain and medulla oblongata stained with Toluidine blue to identify brainstem structures. SC, Superior colliculus; CG, central gray; mRt, mesencephalic reticular formation; III, oculomotor nucleus; RN, red nucleus; V4, fourth ventricle; TSV, spinal trigeminal tract; NSV, spinal trigeminal nucleus; Gi, gigantocellular reticular nucleus; LRt, lateral reticular formation. Scale bar, 4 mm. **(B,E)** Mosaic images of midbrain and medulla oblongata, respectively, from tissue sections adjacent to those shown in **(A,D)**, captured at 10X with an Olympus IX83 microscope. Numerous aminostilbamidine-labeled neurons are observed in the magnocellular part of the contralateral RN, and the ipsilateral Gi. Scale bar, 1 mm. **(C,F)** Confocal fluorescence images of the regions in rectangles in **(B,E)**, showing neurons labeled in the contralateral RN and ipsilateral Gi at higher magnification. Scale bar, 200 μm.

Aminostilbamidine-labeled neurons were also found in brainstem nuclei in both pigs. Again, we focused on pig 1 because the tracer spread mostly in one side (right) of the spinal cord. In this animal, numerous neurons and their cellular processes were filled with the tracer in the contralateral red nucleus and the ipsilateral gigantocellular reticular nucleus (Figure 2). Cellular uptake of the tracer was quite specific, i.e., no spurious cell labeling occurred in the brainstem and rostral CNS structures and as already mentioned, fluorescent cells were found mostly contralaterally in the RN and the cerebral cortex, with only layer-V neurons being fluorescent in the latter, indicating that the tracer was effectively transported through axons projecting to the spinal cord.

Despite we injected the tracer slowly and divided the volume in two tissue depths at each spinal longitudinal coordinate, some aminostilbamidine solution refluxed through the needle path after the injection and spilled into the cerebrospinal fluid. This led to tracer uptake by meningeal and perivascular cells, as well as by cells of microglial morphology, in the spinal cord tissue adjacent to the injection sites. By no means this biased the identification of retrogradely-labeled neurons.

### Distribution of Corticospinal Axons in the Spinal White Matter

**FIGURE 3:**
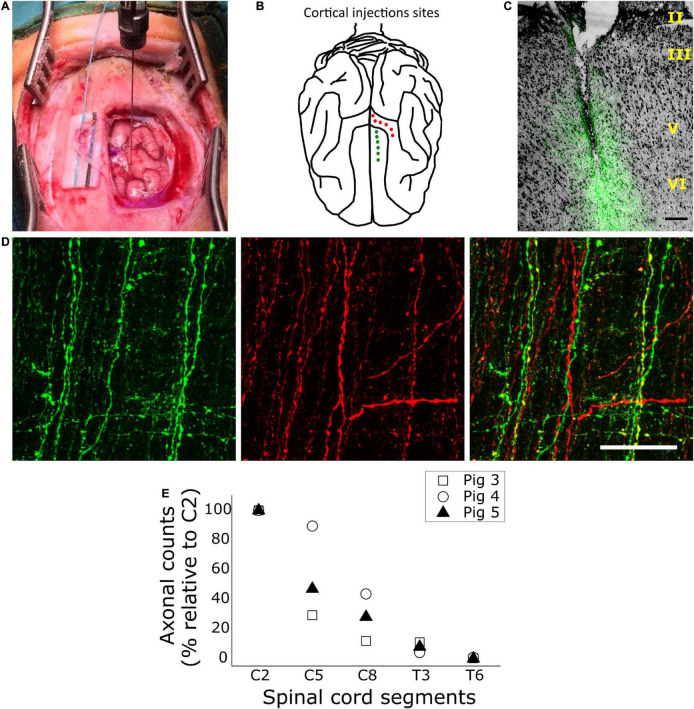
Anterograde tracing of the corticospinal tract (CST). **(A)** Intraoperative photograph of the surgical approach with an injection of tracer being applied in the left cruciate gyrus. **(B)** Illustration depicting the cortical injections sites in the porcine cerebral cortex. Red points illustrate injection sites in the cruciate gyrus and green points illustrate injection sites in the frontal superior gyrus. **(C)** Exemplification of a cortical injection site in the frontal superior gyrus, generated by merging transmitted light (for visualizing Toluidine blue) and fluorescence (for Alexa 488-conjugated dextran). Scale bar, 200 μm. **(D)** Confocal fluorescence images of corticospinal axons in the right dorsolateral funiculus of the C7 spinal cord segment, taken from a horizontal, 50-μm-thick section at ∼2,300 μm of depth in the dorsal-ventral plane. Axons arising from the premotor cortex appear in green, and those from M1 appear in red. Scale bar, 50 μm. **(E)** Quantification of CST axons within the white matter of different spinal cord segments, shown as a percentage relative to axons counted at C2 in the same animal. Data represent axon counts for each pig as indicated in the inset.

Figures 3A,B illustrates the placement of anterograde tracer injections along the cruciate and the frontal superior gyrus, and Figure 3C shows the appearance of an injection site at 2-months later. As expected, the tracer spread mainly over layer V, where CSNs are located, and over layer VI, although the tip of the needle frequently reached the white matter beneath layer VI. CST axons from both M1 and PM showed intense fluorescent signal (Figure 3D) and could be easily identified. Initially, we counted corticospinal axons and examined their distribution along the spinal cord. Because the tracer volume and the survival time were kept constant irrespective of animal age, the older the animal (and presumably the larger the brain) the less axons were labeled in the spinal cord (Supplementary Table 1). Despite inter-individual differences in axonal numbers, the general pattern of axonal distribution was very similar in all subjects. Corticospinal axons from both M1 and PM reached the T6 segment. However, they progressively decreased in number from C2 to T6 (Figure 3E), with most axons (∼92% of those counted at C2) ending rostral to T3.

The largest part of the CST decussated at the pyramids; in average for the three pigs, 84.9 ± 1.5% of axons were found in the contralateral (right) side of the spinal segment C2. In both sides, the axons traveled mainly in three funiculi, namely the DLF, DMF and VMF. However, most corticospinal axons were found in the contralateral DLF (73 ± 4.9%), followed by the ipsilateral DLF (10.4 ± 0.7%), contralateral DMF (8.6 ± 1.8%), contralateral VMF (3.3 ± 1.8%), ipsilateral DMF (3 ± 0.9%), and ipsilateral VMF (1.8 ± 1.1%). We found no significant differences in the distribution of axonal projections from M1 and PM within the spinal WM funiculi. For instance, 73.7 ± 4.7% and 72.2 ± 5.5% of axons from M1 and PM, respectively, were located in the contralateral DLF. Nevertheless, as shown in Supplementary Table 1, the total number of axons labeled after tracer injection in M1 exceeded by 70% the number labeled after injection in PM (*t*-test, *p* < 0.05). Such a difference in the contribution of M1 and PM to the CST was not expected considering the counts of retrogradely-labeled CNSs, which showed a similar or even larger number of CSNs in PM (section “Retrograde Tracing of Encephalic Neurons Projecting to the Spinal Cord”). Therefore, the number of anterogradely-labeled axons might have been biased by a better diffusion or uptake of the Alexa-594-labeled dextran injected in M1, compared to the Alexa-488-labeled dextran injected in PM.

### Corticospinal Innervation of the Spinal Gray Matter

**FIGURE 4:**
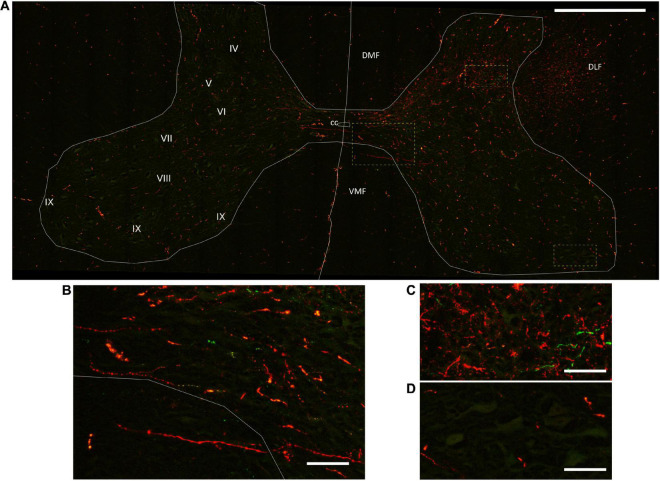
Corticospinal axon distribution in the spinal gray matter laminae. **(A)** Mosaic of confocal fluorescence images acquired with a 20X objective from a 60-μm-thick transverse section of the C6 spinal cord segment, illustrating the innervation of the spinal gray matter by corticospinal axons. The gray matter laminae are an approximation from human and rat descriptions (Paxinos and Watson, 1998; Mai and Paxinos, 2012). Scale bar, 1 mm. **(B–D)** Magnification of the spinal cord regions in the rectangles in A, showing decussating spinal axons **(B)**, as well as the comparative innervation of the intermediolateral **(C)** and ventrolateral **(D)** areas of the gray matter. Scale bar, 100 μm. DMF, dorsomedial funiculus; DLF, dorsolateral funiculus; VMF, ventromedial funiculus; cc, central canal.

Figure 4 exemplifies the topography of corticospinal axons in the WM funiculi and their approximate distribution within the spinal GM laminae. Axons from the DLF entered the GM through laminae V-VII (Figure 4C), whereas axons from the DMF entered by the medial aspect of lamina IV. Axons that traveled in the ipsilateral WM funiculi decussated in all segments of the spinal cord, forming commissural fibers that terminated in the contralateral side, frequently in the same segment of decussation. In general, CST axonal varicosities were more concentrated in laminae IV, V, VI, and VII. A profuse innervation was consistently detected in the IB nucleus, which is located in the medial region of lamina IV from C1 to C6 and contains sensory relay neurons projecting to the thalamus (Granum, 1986; Kemplay and Webster, 1986). Some axonal terminations were also present in lamina VIII, frequently arising from decussating commissural fibers (Figure 4B), and in the ventromedial region surrounding putative motoneurons for axial muscles (Cerro et al., 2021). The finding of corticospinal axons arriving to lateral laminae IX was rare (Figure 4D).

**FIGURE 5:**
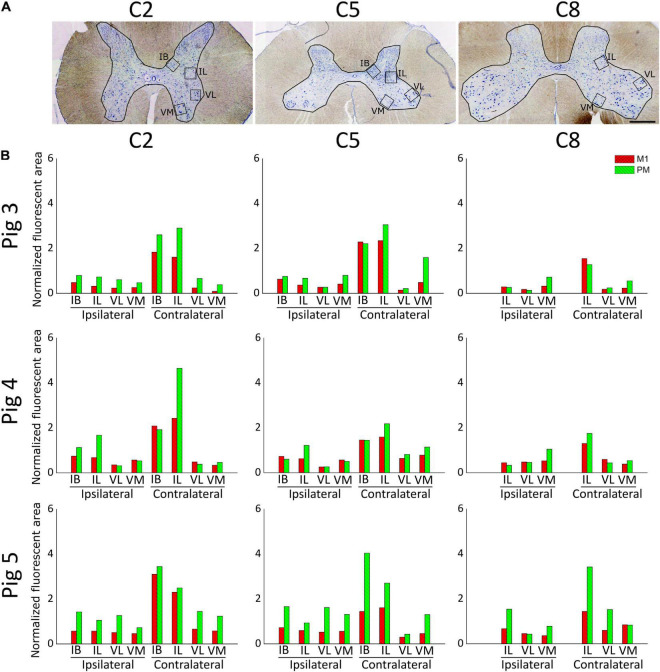
Estimation of spinal gray matter innervation by axons from M1 and premotor cortex (PM) **(A)** C2, C5, and C8 transversal spinal cord sections processed for Toluidine blue staining. Squares illustrate the regions of the gray matter analyzed. IB, internal basilar nucleus; IL, intermediolateral region; VM, ventromedial region; VL, ventrolateral region. Scale bar, 1 mm. **(B)** Gray matter innervation represented as the area occupied by CST axonal branches and endings in the regions illustrated in A for the spinal cord segments C2, C5, and C8, normalized to the amount of CST axons counted in C2. Values from M1-arising axons are shown in red, and those from PM-arising axons appear in green. Three tissue sections were analyzed for each data point.

We performed a qualitative analysis of the innervation of the GM by corticospinal axons in the three anterogradely-traced pigs (4, 5, and 7 months-old at the time of death, respectively). Because the limits of the GM laminae could not be defined precisely in the pig spinal cord, we considered more reliable to focus in the IB, IL, VL and VM regions of the GM (Figure 5A). Figure 5B shows the area occupied by the projections from M1 and PM in the mentioned GM regions at C2, C5 and C8. The gross pattern of GM innervation by CST axons was similar for the three pigs. For all segments, the contralateral side was more abundantly innervated than the ipsilateral side, particularly at the IB and IL regions. M1 and PM axonal projections terminated in the same GM zones.

**FIGURE 6:**
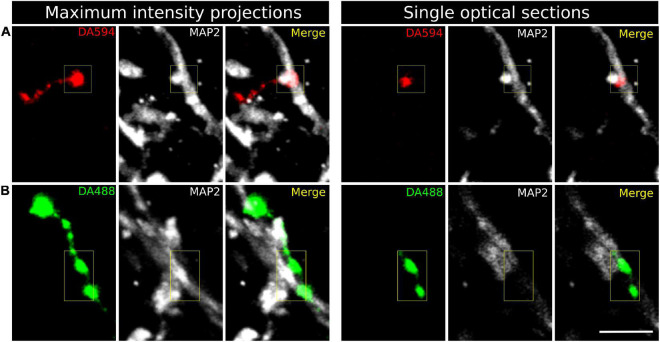
Confocal fluorescence images of corticospinal axon varicosities in the gray matter of the C3 spinal segment. **(A)** Terminal varicosity from an axon arising in M1, labeled with Alexa 594-conjugated dextran (DA594). **(B)** *En passant* axonal varicosities from a terminal branch of a PM-arising axon injected with Alexa 488-conjugated dextran (DA488). Transverse spinal cord sections were processed for microtubule-associated protein 2 (MAP2) immunohistochemistry to visualize neuronal somas and dendrites (white). Maximum intensity projections were obtained from a z-stack of twelve serial optical sections separated 0.84 μm. Scale bar, 5μm.

MAP2 immunohistochemistry was used to label the spinal GM and get preliminary insight into the targets of CST axons. The anti-MAP2 antibody selectively recognized somas and dendrites, with no apparent labeling of axons as revealed by simultaneous neurofilament immunohistochemistry (Supplementary Figure 2). When visualized at high magnification in the confocal microscope, both *en passant* and terminal varicosities of corticospinal axons from M1 and PM were abundant in the spinal laminae IV–VII and most frequently formed axodendritic contacts (Figure 6), with much less presence on neuronal somas. *En passant* and terminal CST axonal varicosities were also juxtaposed to postsynaptic densities as stained by immunohistochemistry for PSD-95 (Supplementary Figure 3), suggesting that the labeled CST axons formed synaptic contacts with spinal cord neurons.

### Cortical Innervation of Brainstem Nuclei

**FIGURE 7:**
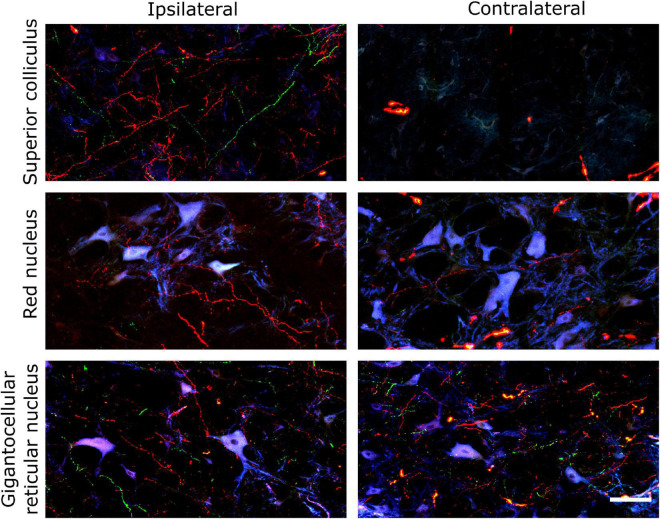
Axonal projections from the porcine cerebral cortex to the midbrain and medulla oblongata. Confocal fluorescence images illustrating M1 and PM innervation of the superior colliculus, red nucleus and gigantocellular reticular nucleus of both sides. The images were captured with a confocal microscope (Leica TCS SP5) using a 20X objective. Neurons are shown in blue taking advantage of their autofluorescence. Scale bar, 100 μm.

**FIGURE 8:**
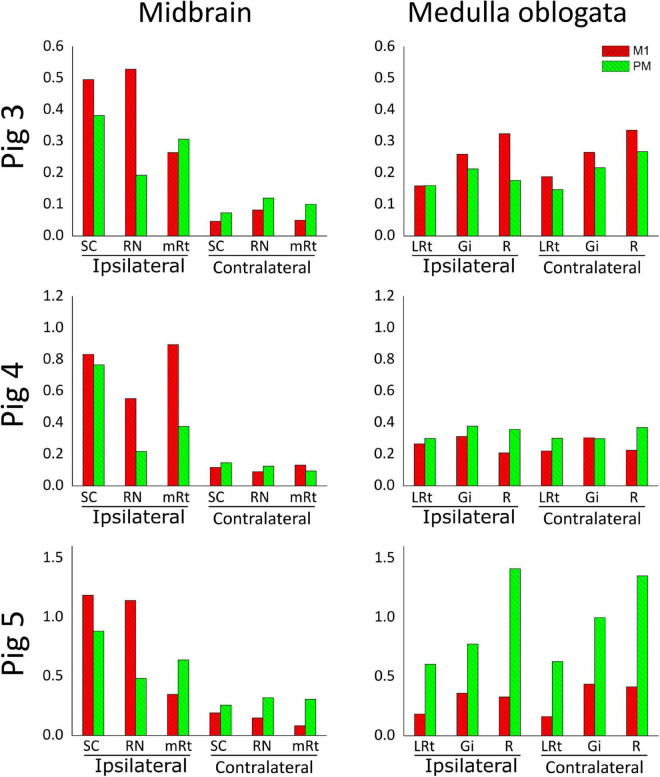
Estimation of brainstem innervation by axons from M1 and PM, represented as the area occupied by CST axonal branches and endings normalized to the amount of CST axons counted in C2. Values from M1-arising axons are shown in red, and those from PM-arising axons appear in green, separately for the side ipsilateral or contralateral to that injected with anterograde neural tracers. SC, Superior colliculus; RN, red nucleus; mRt, mesencephalic reticular formation; LRt, lateral reticular formation; Gi, gigantocellular reticular nucleus; R, raphe nuclei.

The similar distribution and termination of corticospinal axons from M1 and PM along the spinal cord prompted us to investigate the ending of cortical projections to the brainstem. The midbrain and the medulla oblongata were studied because they contain the main brainstem nuclei that receive cortical afferents and provide inputs to spinal neurons (Kuypers, 1962; Armand, 1982; Canedo, 1997). In fact, as described in section “Retrograde Tracing of Encephalic Neurons Projecting to the Spinal Cord” and illustrated in Figure 2, the main motor brainstem nuclei projecting to the spinal cord, including the RN and the Gi, were well labeled by aminostilbamidine injections in caudal C1. As for the spinal cord, we analyzed the three anterogradely-traced pigs for brainstem innervation and several common findings were obtained. Copious branches from M1 and PM axons terminated in the midbrain and the medulla oblongata (Figure 7), with abundant *en passant* and terminal varicosities contacting dendrites and neuronal somas. However, most cortical projections to the midbrain ended ipsilaterally, whereas those to the medulla oblongata were markedly bilateral (Figures 7, 8). In the midbrain, the SC and the RN received the densest innervation, and the RN was innervated mainly by axons arising from M1 (Figures 7, 8). On the contrary, all analyzed nuclei in both sides of the medulla oblongata received substantial innervation from both cerebral cortices (Figure 8). When considering data from the three pigs, no consistent differences were found in the amount of innervation from M1 and PM in the medulla oblongata.

## Discussion

**FIGURE 9:**
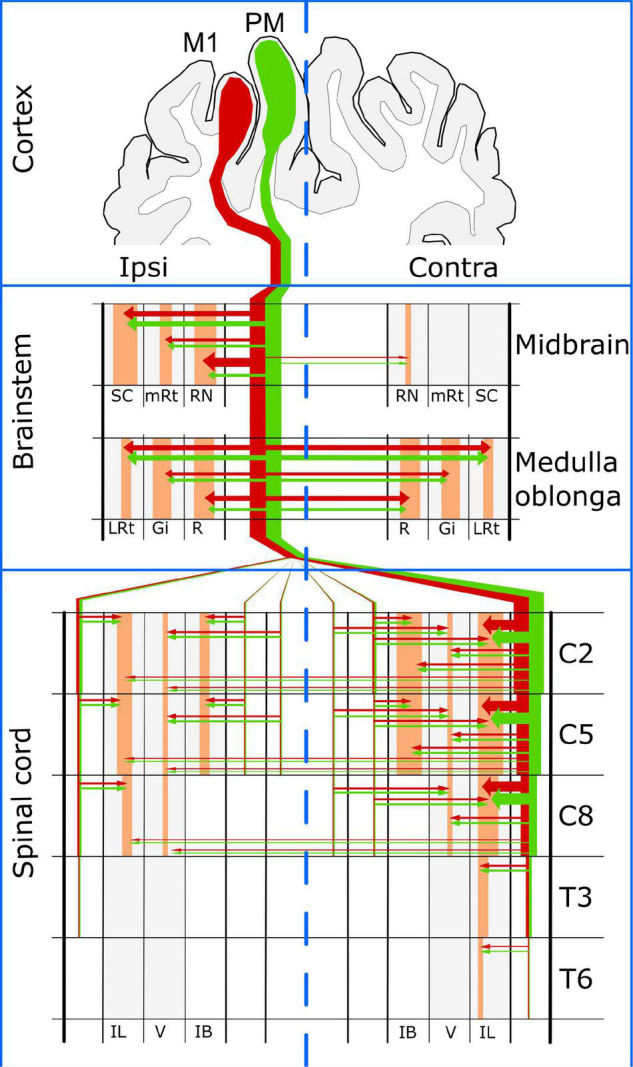
Summary of the cortical motor system in the domestic pig. SC, superior colliculus; RN, red nucleus; mRt, mesencephalic reticular formation; LRt, lateral reticular formation; Gi, gigantocellular reticular nucleus; R, raphe nuclei; IL, intermediolateral gray matter region; V, ventral gray matter region; IB, internal basilar nucleus.

Information about the porcine cortical motor system is scarce. Here we present evidence that at least two cortical regions contribute CST axons in domestic pigs. The same cortical regions also innervate the brainstem nuclei that originate spinal projections, thus allowing the cerebral cortex to command spinal circuits directly through the CST or indirectly through the brainstem (Figure 9). Although the gross distribution of corticofugal axons from both cortical regions was very similar at the level of the medulla oblongata and the spinal cord, the major innervation of the RN arose mainly from M1 and was markedly unilateral as reported for primates. Pigs also showed similitude with primates regarding the location and distribution of CST axons in the spinal WM funiculi and the innervation of GM laminae IV-VIII; however, the tract was apparently limited to the cervical and mid-thoracic spinal cord segments and had almost no axonal endings in lateral lamina IX. These results suggest that the porcine cerebral cortex can directly command foreleg movements through cervical spinal neurons but relies mostly on brainstem nuclei to control hindlimb motor activity. Additionally, innervation of the IB nucleus by the porcine CST provides further support for the role of this tract in the selection of sensory inputs for the coordination of posture and movement. Our data provide an anatomical background for behavioral and physiological sensorimotor research in swine, as well as for using pigs as model of human neurological diseases.

Although we used pre-pubertal animals, we can safely assume that our findings reflect the origin and termination of the corticospinal and cortico-brainstem projections in adult swine. Histological analyzes of the CNS were performed at 4–7 months of born, an age at which the porcine cortical motor system is expected to be mature in terms of neural connectivity. To the best of our knowledge, no information exists regarding the development of porcine motor corticofugal axons; however, ungulates are precocial animals born with nearly mature sensory-motor abilities. Male domestic pigs reach sexual maturity by 7-months of born; therefore, ages of 2-, 5-, and 7-months in pigs are equivalent to about 4-, 10-, and 14-years in humans, respectively (Tohyama and Kobayashi, 2019). Domestic piglets have the adult brain neuronal numbers and sulcal pattern established at birth (Jelsing et al., 2006; Ernst et al., 2018), and show a perinatal brain growth spurt like that of humans (Dickerson and Dobbing, 1967). These facts, together with evidence that the human CST arrives to the lower cervical segments before birth (Eyre et al., 2000), and our observation of axonal Wallerian degeneration in the dorsolateral and ventromedial spinal fascicles, where the porcine CST is located, caudal to a cervical C6 spinal cord hemisection in 2-month-old piglets (Cerro et al., 2021), support the notion that all porcine brain-spinal cord axons have reached their target regions in the perinatal period. On the other hand, M1 thickness increases by 60% during the early postnatal life, but it reaches 95% of its adult value by 5–6 months of age (Desantis et al., 2021). CST myelination likewise occurs in the postnatal period in domestic pigs, reaching a mature state by 6 postnatal months (Fang et al., 2005). Because completion of neural circuit formation and myelination of axonal branches are interrelated events (Wang et al., 2021), no major changes are expected in the healthy cortical motor system connectivity after the first half-year of postnatal life in domestic pigs, except for changes in the number of axonal endings, synaptic maturation, and physiological remodeling associated to learning and neural activity (Martin, 2005). In this regard, development of corticomotoneuronal connections deserves consideration. In rhesus monkeys, the CST arrives to the lower cervical spinal cord and terminates in the contralateral dorsal horn and intermediate zone before birth, but only populates the lateral lamina IX and contacts the motoneurons between birth and 8-postnatal months (Kuypers, 1962; Armand et al., 1997). Increase in the density of axonal branches and terminals within the hand motoneuronal nuclei is still observed during the second postnatal year (Armand et al., 1997). Because male rhesus monkeys reach sexual maturity by 4 years (Mann et al., 1998), 8-months of age in this species is equivalent to ∼1 month in domestic pigs, and 2 years correspond to ∼3.5 months. In humans, corticomotoneuronal connections seem to be established prenatally (Eyre et al., 2000, 2001). Considering the available data from humans, monkeys, and swine together, there is no reason to suspect late (>4 postnatal months) innervation of the lateral cervical motoneurons, or the lumbar spinal cord, by the CST in pigs.

To the best of our knowledge, retrograde tracing of pig CST neurons has not been performed before. Initial studies applying spinal injections of HRP showed that M1, the primary and secondary sensory cortices, and area 5 of the suprasylvian gyrus send corticospinal axons in cats (Coulter et al., 1976; Groos et al., 1978). Nudo and Masterton (1990) retrogradely labeled the cortical neurons that project axons to the spinal cord in several rodents, lagomorphs, and some primates, showing that CST axons arise from two or three neocortical regions in the 22 species studied. More than 90% of corticospinal neurons were observed in the contralateral hemisphere in 18 of the 22 species and, except for rodents, no labeled neurons were observed near the frontal pole. Subsequent studies confirmed the existence of multiple cortical regions that originate CST axons and participate in distinct neural networks within the brain and spinal cord in primates (Dum and Strick, 1991; Galea and Darian-Smith, 1994; Luppino et al., 1994; Strick et al., 2021). In fact, the proliferation of cortical motor areas, together with the emergence of a new M1 region that gives rise to direct corticomotoneuronal synapses, is thought to account for the enhancement of motor capabilities and dexterity in some primates and humans (Strick et al., 2021). Our results show that at least two cerebral cortex regions in swine have a substantial spinal projection despite the low dexterity of the porcine forelimb. Thus, in swine as in other mammals, M1 is not the “final common pathway” of the cerebral cortex to the spinal cord and at least one more cortical region has direct access to the spinal networks, likely enhancing the motor repertoire and the coordination of posture and movement. Although aminostilbamidine likely labeled a fraction of CSNs and might have failed in identifying a cortical region specifically projecting to the lumbar spinal cord, the latter possibility seems unlikely in view of the tissue damage and spread of the tracer in the right spinal GM and WM of pig 1, and of electrophysiological experiments locating the porcine hindleg cortical motor area in the caudal part of the cruciate gyrus (Breazile et al., 1966; Palmieri et al., 1987; Benavides et al., 2017), which was efficiently labeled in our experiments by both retrograde and anterograde neural tracers. However, studies combining anatomical and electrophysiological techniques are required for understanding the influences of the porcine motor cortex on the cervical and lumbar spinal cord.

The precise functional correspondence between the identified porcine cortical motor areas and those of other animals is uncertain. Nevertheless, because of its location, cytoarchitecture and lack of corticomotoneuronal connections, the M1 of pigs seems comparable to the “old” M1 of rodents and primates, which commands motoneuron activity through a disynaptic pathway via interneurons in the intermediate zone of the spinal cord (Rathelot and Strick, 2009; Strick et al., 2021). It may also be equivalent to the “old” M1 of cats, specifically to area 4γ (Hassler and Muhs-Clement, 1964), which lacks a significant direct connection with motoneurons (Lundberg and Voorhoeve, 1962; Porter, 1985), and shows discharge patterns correlated with contralateral forelimb and hindlimb muscle activity (Ghosh, 1997; Fortier-Lebel et al., 2021). The second porcine cortical region we found contributing CST axons was the PM cortex. This area can be distinguished from the prefrontal cortex by a less cell dense layer III, and a wider layer V with more large pyramidal neurons (Jelsing et al., 2006). It is also distinct from M1 that shows a sub-layering of layer III and disperse, very large, cell somata in layer V (Betz giant cells) (Saikali et al., 2010). Based on its spatial location and its innervation of the entire cervical spinal cord, the porcine PM could correspond to the rat M2 or primate PMd cortex (Morecraft et al., 2019; Strick et al., 2021), and to areas 6aα and 6aγ in cats, as delimited by Avendaño et al. (1992), which also project to the cervical spinal cord (Ghosh, 1997). In primates, the premotor cortex has been defined operationally as the regions of the frontal lobe that project directly to M1 (Dum and Strick, 2002). We are not aware of studies investigating the circuitry of the porcine motor cortex; nevertheless, PM and M1 are in proximity and likely have extensive interconnections as in primates (Dum and Strick, 2005). In the latter, each premotor area projecting to the spinal cord participates in specific neural loops with the parietal and prefrontal cerebral cortex, as well as with the basal ganglia and cerebellum, and seems involved in different aspects of motor behavior (Rizzolatti et al., 1998; Dum and Strick, 2002). As discussed below, the main difference we found between M1 and PM in pigs was the corticorubral projection, which arose mostly from M1. However, more detailed studies on the efferent connections, as well as the description of their afferents, are necessary to better understand the functional anatomy of the porcine cerebral cortex.

It is remarkable that PM has not been identified as a source of motor outputs when mapping the porcine motor cortex with stimuli applied through epicortical electrodes (Breazile et al., 1966; Palmieri et al., 1987). This likely reflects the different cytoarchitecture and functional role of PM and M1. It is tempting to speculate that in pigs, as in primates, the largest pyramidal neurons from M1 have the lowest activation thresholds, largest axonal diameters, and fastest axonal conduction velocities, thus providing the most powerful cortical input to spinal neurons when electrically activated (Innocenti et al., 2019). The predominance of smaller pyramidal neurons at PM, with lower axonal diameter and slower conduction velocity (Innocenti et al., 2019; Kraskov et al., 2019) would make more difficult to evoke a motor potential from this region. Nevertheless, activation of different sets of brainstem neurons by M1 and PM, as well as the different physiology of both regions (Kraskov et al., 2019) might also distinctly shape their motor outputs.

Guided by retrograde identification of CST neurons, we could inject anterograde tracers to visualize corticofugal projections from both M1 and PM to the spinal cord and the brainstem. Although the tracer injections likely labeled a limited number of cortical neurons, thousands of CST axons were present at C2 and traveled down to the thoracic spinal cord indistinctly of their cortical origin. These results confirm that pigs have a complete pyramidal system with cortico-bulbar and corticospinal components, in agreement with recent studies in swine that found CST axons descending along the complete cervical spinal cord after injection of anterograde tracers in M1 (Leonard et al., 2017). Our results likewise confirm and extend the suggestion that PM contributes corticospinal axons (Bech et al., 2018). We additionally showed that the CST reaches at least the spinal segment T6, which is consistent with the recording of D waves at T6 after transcranial stimulation of M1 in Yucatan minipigs (Benavides et al., 2017). Although we cannot rule out that some axons innervate segments caudal to T6, it seems unlikely that an important contingent of fibers extends to the porcine lower thoracic or lumbar segments because the CST terminated always in T6 irrespective of the animal size and age. Thus, although the porcine cortical motor system can directly influence the spinal circuits controlling the foreleg, it seems to rely on brainstem nuclei and cervical propriospinal neurons for driving the output of the lumbar spinal cord networks, and hence the execution of hindlimb movements.

Approximately 86% of the CST fibers decussate at the pyramidal level and descend by the contralateral DLF in Gottingen minipigs (Bech et al., 2018). Similarly, we found that 84.9% of axons decussate rostral to C2 in domestic pigs, and that most axons run in the DLF. In addition, a consistent finding was that about 10% of CST axons traveled in the VMF and 5% in the DMF, mostly contralateral, in all anterogradely-traced pigs. Notably, we also observed numerous CST axons decussating across the cervical spinal midline. This distribution of CST axons in the porcine cervical spinal WM resembles more the tract arrangement in primates and humans (Schoen, 1964; Nathan et al., 1990; Rosenzweig et al., 2009; Welniarz et al., 2016) than in rats, which have the main contingent of CST fibers running in the dorsal funiculus (Collazos-Castro et al., 2005; Welniarz et al., 2016). The anatomy of the porcine CST likewise seems to diverge from that of other ungulates, which apparently have a shorter tract restricted to the upper cervical segments, with less decussation, and located in the spinal ventral commissure (Schoen, 1964). Albeit the ungulate CST needs to be re-assessed with modern neuroanatomical techniques, a more developed CST might confer pigs a greater versatility and dexterity in foreleg use compared to goat, cow, sheep, and horse.

We observed no major differences in the distribution of M1 and PM corticospinal fibers, neither in the GM laminae nor along the spinal cord. Although those cortical regions may share some common spinal targets, their specific connectivity needs to be investigated using complementary and more advanced neural tracing techniques. In fact, the combination of CST anterograde tracing, retrograde tracing of spinal neurons with transsynaptic viruses, and electrophysiological techniques is unveiling unique interneurons targeted by subsets of CST axons (Asante and Martin, 2013; Gu et al., 2017; Ueno et al., 2018; Steward et al., 2021), and a similar modular structure of M1 and PM corticospinal circuits is expected to exist in pigs. The gross anatomy and cytoarchitecture of the cervical spinal cord have great resemblance between humans and pigs (Cerro et al., 2021), and therefore the CST axons of each WM compartment likely contact homologous spinal neurons in both species, except for the spinal motoneuron nuclei. As expected for an artiodactyl animal, porcine CST axons barely entered the ventrolateral lamina IX, suggesting that corticomotoneuronal synapses are very scarce. This observation agrees with the concept that the corticomotoneuronal connection developed in parallel with digit dexterity (Kuypers, 1982; Heffner and Masterton, 1983; Porter, 1985; Bortoff and Strick, 1993; Gu et al., 2017). However, porcine M1 and PM seem to have extensive disynaptic access to the cervical spinal motoneurons. The dorsolateral component of the CST mainly innervated the lateral intermediate zone in the cervical spinal cord enlargement, where interneurons that control distal forelimb motoneurons are located. Moreover, numerous axonal varicosities were observed in the same GM region of upper cervical segments, likely targeting propriospinal neurons that project to C6-Th1 (Alstermark et al., 2007; López-Dolado et al., 2013) and are involved in foreleg reaching movements (Isa et al., 2013).

In the upper cervical spinal cord, also the IB nucleus was profusely innervated by CST axons arising from both cortical regions and traveling in the DMF and DLF. This finding is in line with studies showing that the IB nucleus is a target for corticospinal axons in cats (Armand et al., 1985) and rats (Valtschanoff et al., 1993). The IB is continuous rostrally with the cuneate nucleus and receives numerous axonal terminals from both primary and postsynaptic sensory afferents in rats and monkeys (Shriver et al., 1968; Webster and Kemplay, 1987; Cliffer and Giesler, 1989; LaMotte et al., 1991), relaying sensory information to the ventrobasal thalamus (Granum, 1986; Kemplay and Webster, 1986). Therefore, as in several other mammals, the porcine pyramidal system seems to have the necessary connectivity to shape the motor output via brainstem nuclei and premotor spinal neurons, as well as by acting on ascending sensory systems, which provide crucial signals for the coordination of posture and movement (Canedo, 1997).

The pattern of innervation of brainstem nuclei by M1 and PM in pigs is also in concordance with that of other mammals. It is well known that the cortical input to the RN originates mainly from the ipsilateral M1 (Kuypers and Lawrence, 1967; Humphrey et al., 1984; Burman et al., 2000). The RuST, arising in the mRN, is large in primates and carnivores and still larger in ungulates (Schoen, 1964). Rubrospinal axons drive motoneuron activity for distal limb muscles and innervate both the cervical and the lumbosacral spinal cord in rodents, primates, and cattle (Holstege and Kuypers, 1987; Strominger et al., 1987; Burman et al., 2000; Collazos-Castro et al., 2006; Chiocchetti et al., 2006; Basile et al., 2021). Therefore, the cortico-rubro-spinal system may enable volitional control of both foreleg and hindleg movements in pigs. Bilateral innervation of the ponto-bulbar reticular formation also takes place in primates (Keizer and Kuypers, 1989; Fregosi et al., 2017; Darling et al., 2018). However, differences may exist in the amount of M1 and PM axons reaching each reticular nucleus, and this issue needs further investigation using a larger number of pigs and refined quantification of synaptic buttons. Cortico-reticulo-spinal projections subserve integrative roles in locomotion and postural adjustments accompanying limb movements (Lawrence and Kuypers, 1968; Canedo, 1997; Schepens and Drew, 2004; Lemon, 2008), and even participate in skilled hand use (Honeycutt et al., 2013; Esposito et al., 2014; Darling et al., 2018). As in primates (Fregosi et al., 2017), the porcine pyramidal system projected to the raphe nuclei, providing also the possibility of modulating neural activity through serotonergic synapses in the spinal cord. Because the mRN, the reticular formation, and the raphe send axons along the entire spinal cord, the cortical disynaptic pathway to the spinal cord through those brainstem nuclei likely plays a major role in porcine motor behavior, coordinating posture and whole-body movements as well as enabling, in parallel with the CST, some degree of fractionated limb use.

## Data Availability Statement

The original contributions presented in the study are included in the article/Supplementary Material, further inquiries can be directed to the corresponding author/s.

## Ethics Statement

The animal study was reviewed and approved by the Ethical Committee for Animal Research of the Hospital Nacional de Parapléjicos.

## Author Contributions

PC collaborated in surgical procedures and acquired and analyzed the data. ÁR-D-L performed tracer injections. JC-C performed tracer injections and conceived and supervised the study. PC and JC-C wrote the manuscript. All authors read and approved the final manuscript.

## Conflict of Interest

The authors declare that the research was conducted in the absence of any commercial or financial relationships that could be construed as a potential conflict of interest.

## Publisher’s Note

All claims expressed in this article are solely those of the authors and do not necessarily represent those of their affiliated organizations, or those of the publisher, the editors and the reviewers. Any product that may be evaluated in this article, or claim that may be made by its manufacturer, is not guaranteed or endorsed by the publisher.

## Supplementary Material

Supplementary Figure S1 Representative injection sites in the caudal region of the spinal segment C1 of pig 1 **(A)** and pig 2 **(B)**. The upper images show 50-μm tissue sections stained with eriochrome cyanine, useful for identification of the tissue damage caused by the injection procedure and the relatively high volume and concentration (8 μl of 4% aminostilbamidine solution) of the tracer applied at each rostrocaudal coordinate. Neural damage in pig 1 was mostly unilateral and involved the complete right GM, part of the dorsolateral funiculus, the ventromedial funiculus, and the dorsomedial funiculus, where CST axons were located. It also extended to the medial white and gray matter on the left side. Damage was markedly bilateral, and mostly restricted to the gray matter and ventromedial funiculus, in pig 2. Images at bottom correspond to non-stained tissue sections adjacent to those at top, showing the remaining fluorescent aminostilbamidine signal. White matter regions in the lateral funiculus of pig 1 showed intense aminostilbamidine labeling in the side ipsilateral (right, red asterisks), but not contralateral (left, white asterisks) to the injections. Scale bar, 1 mm.

Supplementary Figure S2 Confocal fluorescence images of porcine spinal cord sections processed for neurofilament (NF, green) and microtubule-associated prote2n 2 (MAP2, red) immunohistochemistry, showing the specific labeling of neuronal somas and dendrites by the anti-MAP2 antibody. **(A)** Gray matter of C3 spinal segment. Scale bar, 100 μm. **(B,C)** Magnification of the squares drawn in **(A)**. The maximum intensity projections shown in **(B)** were obtained from a z-stack of nine serial optical sections separated 0.84 μm. Scale bar, 10 μm.

Supplementary Figure S3 Confocal fluorescence images of spinal cord sections processed for postsynaptic density protein 95 (PSD-95) immunohistochemistry, showing putative synaptic contacts by CST axons in the C3 spinal gray matter. Axons arising from M1 appear in red and PSD-95 in blue. **(A)** Maximum intensity projections were obtained from a z-stack of seventeen serial optical sections separated 0.42 μm. **(B–D)** Single optical sections from **(A)**. Scale bar, 5 μm.
