# Supplementary figures and images for: The Cortical Motor System in the Domestic Pig: Origin and Termination of the Corticospinal Tract and Cortico-Brainstem Projections
Source: Front Neuroanat. 2021 Nov 1;15:748050. doi: 10.3389/fnana.2021.748050 (PMC8591036; doi:10.3389/fnana.2021.748050)

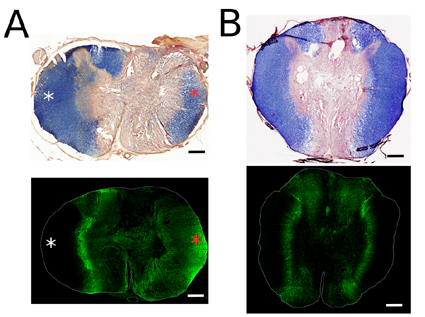

Supplement: Supplementary Figure S1 — Representative injection sites in the caudal region of the spinal segment C1 of pig 1 (A) and pig 2 (B). The upper images show 50-μm tissue sections stained with eriochrome cyanine, useful for identification of the tissue damage caused by the injection procedure and the relatively high volume and concentration (8 μl of 4% aminostilbamidine solution) of the tracer applied at each rostrocaudal coordinate. Neural damage in pig 1 was mostly unilateral and involved the complete right GM, part of the dorsolateral funiculus, the ventromedial funiculus, and the dorsomedial funiculus, where CST axons were located. It also extended to the medial white and gray matter on the left side. Damage was markedly bilateral, and mostly restricted to the gray matter and ventromedial funiculus, in pig 2. Images at bottom correspond to non-stained tissue sections adjacent to those at top, showing the remaining fluorescent aminostilbamidine signal. White matter regions in the lateral funiculus of pig 1 showed intense aminostilbamidine labeling in the side ipsilateral (right, red asterisks), but not contralateral (left, white asterisks) to the injections. Scale bar, 1 mm. [file Image_1.TIF]

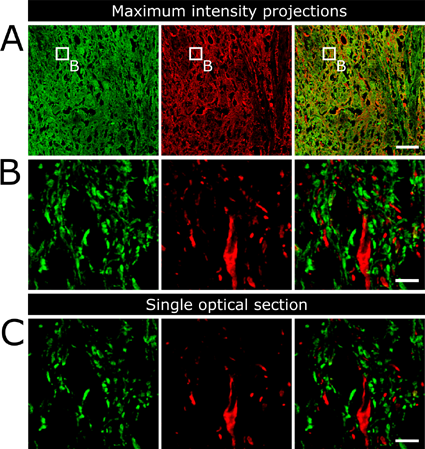

Supplement: Supplementary Figure S2 — Confocal fluorescence images of porcine spinal cord sections processed for neurofilament (NF, green) and microtubule-associated prote2n 2 (MAP2, red) immunohistochemistry, showing the specific labeling of neuronal somas and dendrites by the anti-MAP2 antibody. (A) Gray matter of C3 spinal segment. Scale bar, 100 μm. (B,C) Magnification of the squares drawn in (A). The maximum intensity projections shown in (B) were obtained from a z-stack of nine serial optical sections separated 0.84 μm. Scale bar, 10 μm. [file Image_2.TIF]

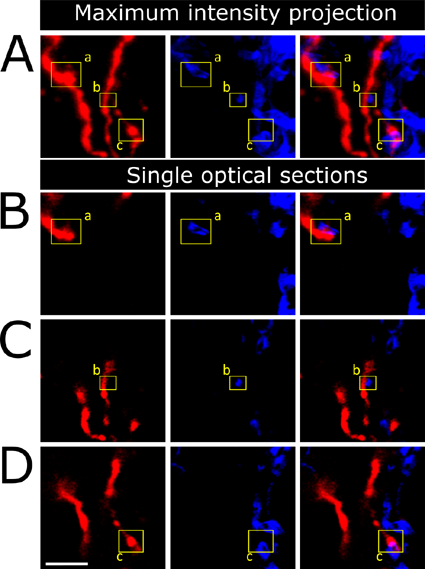

Supplement: Supplementary Figure S3 — Confocal fluorescence images of spinal cord sections processed for postsynaptic density protein 95 (PSD-95) immunohistochemistry, showing putative synaptic contacts by CST axons in the C3 spinal gray matter. Axons arising from M1 appear in red and PSD-95 in blue. (A) Maximum intensity projections were obtained from a z-stack of seventeen serial optical sections separated 0.42 μm. (B–D) Single optical sections from (A). Scale bar, 5 μm. [file Image_3.TIF]
