# Supplementary material for: The Cortical Motor System in the Domestic Pig: Origin and Termination of the Corticospinal Tract and Cortico-Brainstem Projections
Source: Front Neuroanat. 2021 Nov 1;15:748050. doi: 10.3389/fnana.2021.748050 (PMC8591036; doi:10.3389/fnana.2021.748050)
Supplement: Supplementary file 4 [file Table_1.docx]

|  |  |  | Ipsilateral | | | Contralateral | | | Total |
| --- | --- | --- | --- | --- | --- | --- | --- | --- | --- |
|  |  |  | DLF | DMF | VMF | DLF | DMF | VMF |  |
| C2 | Pig 3 | M1 | 209 | 86 | 34 | 1170 | 196 | 119 | 1814 |
|  |  | PM | 151 | 55 | 18 | 788 | 175 | 65 | 1252 |
|  |  | Total | 360 | 141 | 52 | 1958 | 371 | 184 | 3066 |
|  | Pig 4 | M1 | 56 | 12 | 17 | 527 | 49 | 30 | 691 |
|  |  | PM | 49 | 5 | 23 | 289 | 25 | 13 | 404 |
|  |  | Total | 105 | 17 | 40 | 816 | 74 | 43 | 1095 |
|  | Pig 5 | M1 | 37 | 10 | 0 | 311 | 29 | 0 | 387 |
|  |  | PM | 6 | 2 | 0 | 41 | 1 | 0 | 50 |
|  |  | Total | 43 | 12 | 0 | 352 | 30 | 0 | 437 |
| C5 | Pig 3 | M1 | 133 | 14 | 24 | 614 | 67 | 40 | 892 |
|  |  | PM | 83 | 7 | 14 | 395 | 23 | 28 | 550 |
|  |  | Total | 216 | 21 | 38 | 1009 | 90 | 68 | 1442 |
|  | Pig 4 | M1 | 36 | 2 | 5 | 473 | 7 | 4 | 527 |
|  |  | PM | 40 | 4 | 1 | 388 | 15 | 4 | 452 |
|  |  | Total | 76 | 6 | 6 | 861 | 22 | 8 | 979 |
|  | Pig 5 | M1 | 3 | 0 | 0 | 105 | 10 | 1 | 119 |
|  |  | PM | 1 | 0 | 0 | 8 | 0 | 0 | 9 |
|  |  | Total | 4 | 0 | 0 | 113 | 10 | 1 | 128 |
| C8 | Pig 3 | M1 | 71 | 6 | 0 | 450 | 46 | 6 | 579 |
|  |  | PM | 30 | 3 | 0 | 230 | 13 | 7 | 283 |
|  |  | Total | 101 | 9 | 0 | 680 | 59 | 13 | 862 |
|  | Pig 4 | M1 | 9 | 0 | 0 | 267 | 7 | 6 | 289 |
|  |  | PM | 8 | 1 | 2 | 165 | 8 | 5 | 189 |
|  |  | Total | 17 | 1 | 2 | 432 | 15 | 11 | 478 |
|  | Pig 5 | M1 | 3 | 0 | 0 | 45 | 0 | 0 | 48 |
|  |  | PM | 0 | 0 | 0 | 4 | 0 | 0 | 4 |
|  |  | Total | 3 | 0 | 0 | 49 | 0 | 0 | 52 |
| T3 | Pig 3 | M1 | 9 | 0 | 0 | 148 | 4 | 5 | 176 |
|  |  | PM | 3 | 0 | 1 | 59 | 2 | 3 | 68 |
|  |  | Total | 17 | 3 | 3 | 207 | 6 | 8 | 244 |
|  | Pig 4 | M1 | 4 | 0 | 0 | 23 | 0 | 0 | 27 |
|  |  | PM | 1 | 0 | 0 | 16 | 0 | 1 | 18 |
|  |  | Total | 5 | 0 | 0 | 39 | 0 | 1 | 45 |
|  | Pig 5 | M1 | 2 | 0 | 0 | 40 | 1 | 1 | 43 |
|  |  | PM | 2 | 0 | 0 | 2 | 1 | 1 | 5 |
|  |  | Total | 4 | 0 | 0 | 42 | 2 | 2 | 48 |
| T6 | Pig 3 | M1 | 0 | 0 | 0 | 2 | 0 | 0 | 2 |
|  |  | PM | 0 | 0 | 0 | 0 | 0 | 0 | 0 |
|  |  | Total | 0 | 0 | 0 | 2 | 0 | 0 | 2 |
|  | Pig 4 | M1 | 0 | 0 | 0 | 9 | 0 | 0 | 9 |
|  |  | PM | 0 | 0 | 0 | 0 | 0 | 0 | 0 |
|  |  | Total | 0 | 0 | 0 | 9 | 0 | 0 | 9 |
|  | Pig 5 | M1 | 0 | 0 | 0 | 0 | 0 | 0 | 0 |
|  |  | PM | 0 | 0 | 0 | 1 | 0 | 0 | 1 |
|  |  | Total | 0 | 0 | 0 | 1 | 0 | 0 | 1 |

**Supplementary Table 1.** Quantification of axons traveling within white mater from M1 and premotor cortex (PM) in dorsolateral funiculus (DLF), dorsomedial funiculus (DMF) and ventromedial funiculus (VMF) to ipsilateral and contralateral side.
